# Regulation of Naturally Acquired Mucosal Immunity to *Streptococcus pneumoniae* in Healthy Malawian Adults and Children

**DOI:** 10.1371/journal.pone.0051425

**Published:** 2012-12-17

**Authors:** Sarah J. Glennie, Dominic Banda, Wakisa Mulwafu, Rose Nkhata, Neil A. Williams, Robert S. Heyderman

**Affiliations:** 1 Malawi-Liverpool-Wellcome Trust Clinical Research Programme, University of Malawi College of Medicine, Blantyre, Malawi; 2 Clinical Group, Liverpool School of Tropical Medicine, Liverpool, United Kingdom; 3 Department of Surgery, University of Malawi, College of Medicine, Blantyre, Malawi; 4 Cellular and Molecular Medicine, School of Medical Sciences, University of Bristol, Bristol, United Kingdom; Facultad de Medicina, Uruguay

## Abstract

Worldwide, invasive pneumococcal disease caused by *Streptococcus pneumoniae* is most common in young children. In adults, disease rates decline following intermittent colonization and the acquisition of naturally acquired immunity. We characterized mucosal and systemic pneumococcal-specific T-cell responses in African children and adults who contend with intense rates of colonization, up to 100% and 60% respectively. We find most Malawian children have high pneumococcal-specific T-cell responses in tonsil tissue and peripheral blood. In addition, frequent commensalism generates CD25^hi^ (Tregs) which modulate mucosal pneumococcal-specific T-cell responses in some children and ≥50% of adults. We propose that immune regulation may prolong pneumococcal colonization and predispose vulnerable individuals to disease.

## Introduction

In sub-Saharan Africa and elsewhere, invasive pneumococcal disease (IPD) is most common in young children, the elderly and immunocompromised [1]. In healthy adults, pneumococcal colonisation and disease declines due to the acquisition of naturally acquired immunity, generated by carriage of the pneumococcus in the nasopharynx [2], [3]. Limiting pneumococcal carriage at the mucosa, due to vaccination, is thought to reduce invasion and result in herd immunity by blocking person-to-person spread, thus protecting those who remain immunologically susceptible.

The immune processes that control colonization and invasion are not well understood. Natural immunity to the pneumococcus was thought to be largely mediated by antibodies to the polysaccharide capsule [2]. However, more recently, animal models and studies of human carriage have implicated a range of sub-capsular protein antigens in protection against both nasal colonization and invasive disease [3]. Furthermore, pneumococcal carriage experiments comparing antibody-deficient with T cell-deficient mice suggest that CD4 T-cells are key to the mucosal clearance of *S. pneumoniae* and that antibodies to pneumococcal antigens correlate with, but may not be required for protection against colonization [4]. In healthy Gambian adults, we found high numbers of effector and central memory CD4 T-cells to pneumococcal antigens in blood [5] and have demonstrated in Malawian adults that the lung mucosal surface is dominated by pneumococcal-specific memory CD4^+^CD45RA^−^CCR7^−^effector cells [6]. More recently in a low carriage intensity UK population, we described an age related acquisition of mucosal T cell-mediated pneumococcal immunity in the nasopharynx, the site of immune induction [7].

We have proposed that in many African populations, susceptibility to IPD is considerably amplified by mucosal immune dysregulation, mediated by environmental, nutritional and microbial pressures including measles, malaria and HIV [8]. In several developing countries, pneumococcal carriage occurs very early in life, reaching up to 100% in children and 60% in adults [9], far exceeding industrialized countries. The impact of intense colonization on the development of natural immunity in this setting is unknown. Recently, we have shown that antigen-specific CD25^hi^ cells regulate mucosal pneumococcal-specific responses in adults in a UK setting of low carriage intensity [7]. As pneumococcal colonization generates low-level inflammation, immune regulation can limit mucosal damage but inadvertently suppress immunity and prolong bacterial colonization. We hypothesize that in sub-Saharan Africa high levels of carriage leads to the generation of large numbers of pneumococcal-specific CD4 T-cells which are then rapidly controlled by localised regulatory T-cells (Treg). Over-regulation may hinder naturally acquired immunity and increase IPD in susceptible individuals.

To address this possibility, we have investigated the acquisition of pneumococcal-specific immunity in the mucosal palatine tonsils and the recirculation of CD4 T-cells in the blood of Malawian children and adults. We also evaluated whether T regs were generated following natural exposure.

## Materials and Methods

### Ethics Statement

The collection of samples and the research performed was undertaken in accordance with institutional guidelines and approved by the University of Malawi College of Medicine Ethics Committee (P.03/10/919) and The Liverpool School of Tropical Medicine Research Ethics Committee (10.60). A total of forty Malawian children and adults were recruited into the study following informed consent.

### Subjects

Palatine tonsils and blood samples were obtained from healthy individuals (aged 1–39 years) undergoing routine tonsillectomy for recurrent tonsillitis or upper airway obstruction at Queen Elizabeth Central Hospital, Mwaiwathu and Seventh Day Adventist Hospitals in Blantyre, Malawi. Tonsils were collected into RPMI media (Invitrogen, Paisley, UK,) supplemented with 100 U/ml penicillin, 100 µg/ml streptomycin (Sigma, Dorset, UK) and blood samples into sodium heparin tubes. Patients with HIV or serious infections were excluded from the study. Due to limited volumes of blood obtained from some subjects, not all assays were performed on every subject.

### Antigens

Pneumococcal culture supernatants (pneumoCCS) were prepared from a standard encapsulated type 2 (D39) *S pneumoniae* strain and an isogenic pneumolysin-deficient mutant (Ply-). Bio-Rad protein assay was used to measure the concentration of pneumoCCS. The culture supernatants were heat inactivated at 56°C for 30 min to reduce toxic effects of pneumococcal proteins. *M. tuberculosis* (MTB) purified protein derivative (PPD RT49) was obtained from Statens Serum Institut, Denmark. Influenza antigens were derived from dialyzed inactive trivalent split virion influenza vaccine (Enzira® 2006/2007) obtained from Aventis-Pasteur, France.

### Cells and Reagents

Blood and tonsil tissue mononuclear cells (MNC) were isolated by histopaque density gradient separation [10], [11]. MNC were harvested, washed in HBSS at 400 g for 10 min, and resuspended in complete RPMI (RPMI 1640 with 100 U/ml penicillin, 0.1 mg/ml streptomycin, 4 mM _L_-glutamine, and 10 mM HEPES buffer). MNC were counted using 0.4% (wt/vol) trypan blue (Sigma), reconstituted in complete RPMI at a concentration of 1×10^6^ cells/ml; with a final concentration of 2% (vol/vol) heat-inactivated human AB serum (National Blood Services, Blantyre) and incubated at 37°C in presence of 5% CO_2._

### Tonsillar MNC CD25^hi^ Cell Depletion

CD25^hi^ cells were depleted from 0.5–1×10^8^ tonsil MNC using anti-human CD25 coated MACS microbeads (Miltenyi Biotec, Surrey, UK) and magnetic cell sorting (MACS) on LD columns (Miltenyi Biotec) according to the manufacturer’s instructions [7], [10]. Purity of depleted cells were typically >96% as assessed by flow cytometry using APC-labeled anti-human CD25 antibody (BD Bioscience, Oxford, UK). Approximately 4–10% of tonsil CD4 T-cells were CD25^hi^. The removal of CD25^hi^ cells has been validated by our group and others as an experimental approach that removes a population of regulatory cells [7], [10], [12].

### Proliferation Assay

As previously [11], MNC were labeled with 2.5 µm carboxyfluorescein diacetate 5,6 succinimidyl ester (CFSE) dye (Invitrogen) to identify dividing T cells. Labeled cells were cultured at 1×10^6^ cells/ml in a 48 well plate with either pneumoCCS (8 µg/ml), Ply- CCS (8 µg/ml), influenza (0.9 µg/ml), MTB-PPD (10 µg/ml) or media only, for 8 days. Cells were harvested and stained with anti-CD4-APC (BD Biosciences) and assessed by flowcytometry. For flowcytometric assays, 10,000–60,000 events were acquired on either FACSCalibur (BD Biosciences, UK) or CyAnADP 9 Color (Beckman Coulter, USA) flowcytometers. Analysis was performed with FlowJo (TreeStar, Ashland, OR). In tonsil and peripheral blood cultures CD4 T cell proliferation from media only was subtracted from antigen-specific responses and then expressed as the percentage of CD4+cells.

### Statistics

Age related acquisition of naturally acquired immunity to upper respiratory tract (URT) pathogens was modeled using non linear regression and fitted with second order polynomial. The Spearmans rank test was used to determine the relationship between mucosal and circulating antigen-specific T-cells. The Wilcoxon signed rank test was used to test differences between undepleted tonsillar MNC and CD25 depleted groups following antigenic stimulation. All analysis was performed using Graphpad Prism5 software and p<0.05 was considered statistically significant.

## Results

### Mucosal Tonsillar and Peripheral Blood Pneumococcal-specific CD4 T-cells are Present in Malawian Children Early in Life

**Figure 1:**
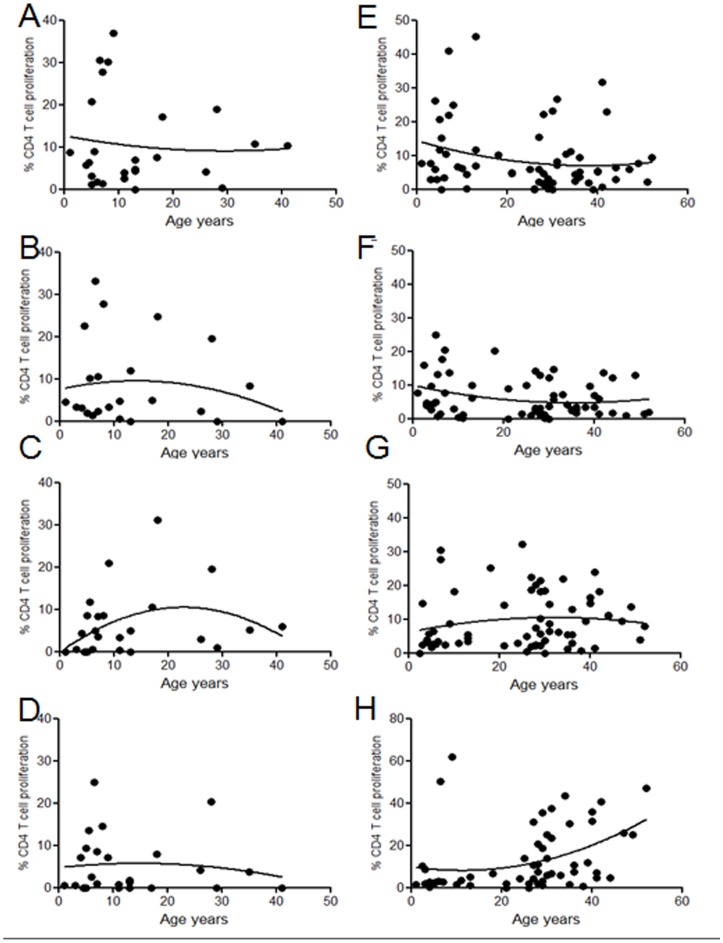
Mucosal and peripheral blood CD4 T cell responses to pneumococcal, influenza and MTB-PPD antigens in Malawian children and adults. Mucosal CD4 T cell proliferation in response to pneumoCCS n = 26 (**a**) and Ply-CCS n = 24 (**b**) influenza n = 25 (**c**) and MTB n = 25 (**d**) in children and adults. Circulating CD4 T cell proliferation in response to pneumoCCS n = 66 (**e**), Ply-CCS n = 67 (**f**), influenza n = 65 (**g**) and MTB-PPD n = 58 (**h**) in children and adults. Data was analyzed by nonlinear regression modeling, fitted with second order polynomial.

Recently we have shown robust CD4 T cell responses to *Streptococcus pneumoniae* in peripheral blood of healthy Malawian children [13] and adults [11], however, the kinetics of naturally acquired immunity with age have not been described. Mucosal tonsillar and peripheral blood CD4 T-cell proliferation was evaluated in response to pneumococcal, influenza and MTB-PPD antigens in children (age 1–12 years) and older children and adults (≥13–41). For analysis purposes these ages were chosen as 1–12 yrs is the age at which the highest rates of pneumococcal colonization occur in an industrialized and resource poor setting [14] before the decline in colonization seen in adulthood. In the mucosal compartment, children had a broad range of pneumococcal-specific tonsillar CD4 T cell responses that were greater in the presence of the immunodominant antigen pneumolysin (WT median 6.3[1.1–37] vs. ply- 3.4[0.5–33.1] (**Fig.1a&b**). WT pneumococcal responses were greater as compared to influenza (median 3.5[0–20.9] (**Fig.1c**) and MTB-PPD (median 2.6[0–24.9] (**Fig.1d**)within the mucosa of children ≤12 years. In individuals aged 13–41 yrs T-cell responses to all antigens were sustained (WT median 6.9[0–19], ply- 3.7[0–24.7], influenza 5.6[0–31.2] and MTB-PPD 1.5[0–20.2]). In the periphery, pneumococcal-specific (**Fig.1e, f**) T-cells were present in the majority of children early in life (WT median 7.6[0–41], ply- 4.8[0.1–24.8] at a comparable level to the mucosa and once again were greater than influenza-specific (**Fig.1g**) and MTB PPD responses (**Fig.1h**) (influenza 3.7[0–30.4], MTB-PPD 2.7[0.4–62.1]). Whilst T cell immunity to the pneumococcus decreased slightly in individuals aged 13–41 yrs ((WT median 5.2[0–45.1], ply- 3.2[0–20.2]) responses to influenza and MTB-PPD increased with age (influenza 7.9[0–32.2], MTB-PPD 7.6[0–46.9]. In both children and adults, high CD4 T-cell responses in peripheral blood did not necessarily correlate with high responses within the mucosa. There was a positive correlation regarding the magnitude of influenza responses in blood and tonsils r = 0.5, p = 0.02 but no correlation for pneumococcal or MTB-PPD responses (data not shown).

**Figure 2:**
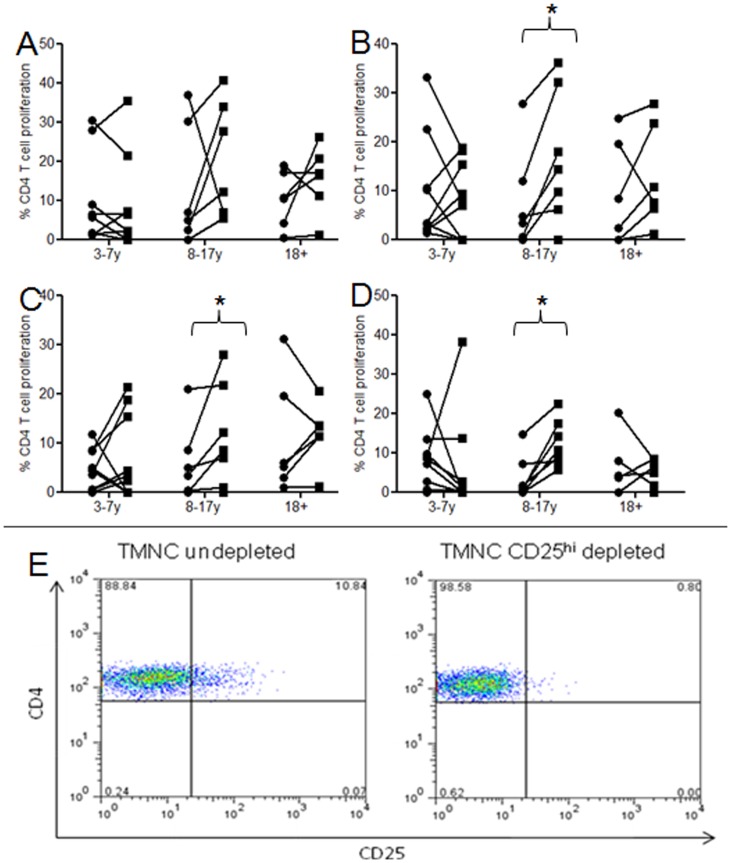
Inhibition of pneumococcal, influenza and MTB responses by regulatory T cells in young children and onwards. Mucosal CD4 T cell responses to pneumoCCS (**a**) and PlyCCS (**b**) influenza (**c**) and MTB PPD (**d**) by CD25^hi^ regulatory T cells in subjects above 3 years old as indicated by increased cell proliferation following depletion of CD25^hi^ cells from tonsillar MNC population. Subjects (n = 22) were grouped into those aged 3–7 yrs, 8 to 17 yrs and >18 yrs. Individual subject’s proliferative response pre (**circle**) and post CD25^hi^ cell depletion (**square**) are shown with a connecting lines. Data was analyzed using Wilcoxon signed rank test, * = p<0.05. Representative flow cytometric plot of CD25 undepleted and depleted tonsillar mononuclear cells (TMNC) (**e**).

### Immune Regulation of Pneumococcal-specific CD4 T-cell Responses in the URT Mucosa of Healthy Malawian Children and Adults

Immune regulation is a common outcome for colonizing bacteria [10]. In a setting of low colonization we and others have shown that mucosal responses to pneumococcal proteins but not influenza antigens are regulated by CD25^hi^ cells in the majority of UK adults [7], [10] and in children [12]. We investigated whether Tregs were generated in Malawian children following intense pneumococcal exposure and whether immune modulation can partly explain prolonged nasopharyngeal colonization and higher incidence of IPD. For this study the age groups used were based on previous work undertaken in an industrialised setting [7], [12] so that the frequency of mucosal regulation could be compared between an area of low pneumococcal colonization vs high colonization. In children aged 3–7 years, we find that 2 out of 8 had increased tonsillar T-cell proliferation to pneumoCCS (**Fig.2a**) after removing CD25^hi^ cells. T-cell responses to Ply-CCS increased in 4 of 9 individuals (**Fig.2b**) influenza-specific responses increased in 6 of 9 individuals (**Fig.2c**) and 1 of 9 in response to MTB-PPD (**Fig.2d**). In older children aged 8–17, we found a significant increase in proliferative responses to Ply-, influenza and MTB-PPD antigens, furthermore in those aged 8–17 and adults ≥18 years, immune regulation was present in ≥50% of those responding to pneumococcal, influenza and MTB-PPD antigens within the URT mucosal tonsillar tissue. Immune suppression was not pneumolysin dependent as regulation was also observed in pneumolysin-deficient cultures.

## Discussion

In an African population with high pneumococcal carriage, we find the majority of young children have high numbers of pneumococcal-specific CD4 T-cells within the URT mucosa and circulation. This contrasts with our observations in lower carriage populations where the acquisition of pneumococcal immunity was more gradual; where mucosal T-cells increased with age and peaked aged 13–21 [7]. Furthermore, in comparison to UK populations, we found far less compartmentalization of pneumococcal and influenza-specific T-cells in mucosal tonsillar tissue versus blood. This rapid acquisition of T-cell memory in this African population appears to be associated with a higher degree of regulation at an earlier age and to a broader range of pathogens than seen in a UK population [7], [10]. To what extent this regulation is protective or maladaptive needs further investigation.

Mucosal immune regulation is a common outcome for other colonizing bacteria [10]. Recently in a UK setting of low pneumococcal colonization, we have shown that CD25^hi^ cells contain a regulatory population that suppress T-cell immunity to *S. pneumoniae* [7] but not to the obligate pathogen influenza. In an area of high colonization and pathogen exposure, we show that depletion of CD25^hi^ cells from tonsillar mononuclear cells increased T-cell responses to all URT antigens, revealing the proliferative capacity of an otherwise regulated population. It could be argued that the removal of CD25^hi^ cells depletes poorly proliferative Tregs and enriches effector T cell proliferative responses. However, we do not think this explains the increase in T cell proliferation seen in our study as we would expect a consistent increase in proliferative response following CD25^hi^ depletion in all ages and for all antigens in a setting of high antigen exposure. Therefore, we propose that our data shows that immune regulation to URT pathogens is more common and broad in Malawian children ≤17 years, with 50% and 56% of individuals showing regulation of pneumococcal and influenza-specific responses respectively, compared to 25% and 18% of children from the UK. The observation that some individuals show immune regulation whilst others do not is expected in human studies, however this variation may be influenced by the duration and density of the latest pneumococcal carriage event. Contrary to our expectations we observed comparable pneumococcal, influenza and MTB-PPD CD4 T-cells in both the mucosa and circulation of Malawian adults, showing no distinct compartmentalization of immune responses to URT pathogens. These findings suggest that high antigen exposure in the URT results in the spillover and recirculation of antigen-specific T-cells that are easily detected in blood. These differences are likely to be driven by the frequency and perhaps density of antigen exposure, in Malawian adults rates of pneumococcal colonization are between 16–20%, whilst the UK is ≤10%.

We show that pneumococcal responses at the mucosal surface are highly regulated in African children; over-regulation may prolong colonization, increase the chance of person-to-person spread and delay herd immunity. Whilst capsular polysaccharide-protein conjugate vaccines (PCV) are effective in reducing colonization and invasive pneumococcal disease of vaccine serotypes, they are less effective in preventing mucosal disease (pneumonia or otitis media) [15]. Also in vulnerable HIV populations, PCV7 was shown to reduce recurrent IPD but immune protection was short-lived [16]. Several pneumococcal surface-expressed proteins, including pneumolysin have been identified as potential vaccine candidates that could compliment the capsular polysaccharide approach. We believe that defining natural mucosal immunity to the pneumococcus is important for the future evaluation of pneumococcal vaccine induced immunity within the mucosal compartment. Recently parenteral meningococcal B vaccination was shown to reprogram pre-existing antigen-specific mucosal responses [17]. Changes in the mucosal Th1/Th2 balance and increased mucosal IL-10 secretion revealed in the study highlight a need for caution when applying parental protein-vaccines to the prevention of disease caused by URT commensals. This is an important consideration when vaccinating HIV-infected individuals as regulatory T-cells are frequently over-represented in the periphery [11], the same may be true within the URT mucosa. As immune dysregulation occurs in many sub-Saharan vulnerable populations [8] we propose that new protein vaccine candidates are screened to reduce the chance of further immunomodulation of pneumococcal responses.
